# Loss of MADD expression inhibits cellular growth and metastasis in anaplastic thyroid cancer

**DOI:** 10.1038/s41419-019-1351-5

**Published:** 2019-02-13

**Authors:** Shikha Saini, Lakshmi Sripada, Kiara Tulla, Prabhakaran Kumar, Fei Yue, Nicholas Kunda, Ajay V. Maker, Bellur S. Prabhakar

**Affiliations:** 1 Department of Microbiology and Immunology, University of Illinois at Chicago, Chicago, IL 60612 USA; 2 Department of Surgery, University of Illinois at Chicago, Chicago, IL 60612 USA; 3 Jesse Brown VA Medical Center, Chicago, IL 60612 USA

## Abstract

Anaplastic Thyroid Cancer (ATC) is an aggressive malignancy with limited therapeutic options and dismal patient survival. We have previously shown MADD to be differentially overexpressed in multiple cancer histologies and to contribute to tumor cell growth and survival. Therefore, we targeted MADD by gene silencing, explored its effect on cellular proliferation and metastases and examined its therapeutic potential in an orthotopic ATC model in athymic nude mice. When compared to untreated control and scramble siRNA, MADD siRNA treatment inhibited the proliferative capacity of 8505C, C643 and HTH7 cells in vitro and 8505C-derived-orthotopic tumor growth in vivo. MADD ablation caused a significant reduction in cellular migration and invasion potential; clonogenic capacity; as well as, mitochondrial length and potential in vitro. This MADD siRNA-induced anti-migratory/invasive effect corresponded with inhibition of epithelial–mesenchymal transition (EMT) and Wnt signaling. Mechanistically, MADD siRNA inhibited TNFα induced activation of pERK, pGSK3β and β-catenin, suggesting that MADD knockdown might exert its anti-migratory/invasive effects, by blocking TNFα/ERK/GSK3β axis. MADD siRNA can inhibit β-catenin nuclear translocation and consequently, the expression of its target genes in ATC cells. In in vivo experiments, along with tumor regression, MADD siRNA treatment also decreased evidence of lung metastases. Immunohistochemically, MADD siRNA-treated tumor tissues exhibited a reduction in Ki67 and N-Cadherin expression, and an increase in E-Cadherin expression. In conclusion, we show the crucial role of MADD in ATC tumorigenesis and metastasis and its potential implications as a molecular target for ATC therapy.

## Introduction

Thyroid Cancer is the most common endocrine malignancy, accounting for 53,990 estimated cases in the USA in 2018^1^. Anaplastic Thyroid Cancer (ATC) constitutes only 1–2% of thyroid cancers, but it disproportionately causes up to 40% thyroid cancer-related deaths^2^. ATC treatment entails an extensive multimodal approach including surgery, adjuvant radiotherapy, and chemotherapy (targeted inhibitors, multi-kinase inhibitors, and genotoxic compounds) with sub-optimal success^3^. About 90% ATC patients invariably present with the un-resectable tumors at the time of diagnosis and with tumor resections having high tumor recurrence rates, forcing this patient population to rely on palliative treatments^2^. Thus, it is imperative to understand the ATC pathogenesis to improve the therapeutic management of ATC patients.

We had previously reported a differentially overexpressed splice variant of IG20 gene, MADD (MAPK-activating Death Domain activating protein) in cancer cell survival in the context of TNFα signaling^4–6^. MADD essentially plays a survival-promoting role against TNFα mediated apoptosis, by explicitly activating MAPKs through Grb2 and Sos1/2 recruitment, followed by activation of ERK without any apparent effect on p38, Jun, and NFκB^5^. It is important to note that TNFα is a multifunctional cytokine and is engaged in other cancer-related processes such as migration, invasion and angiogenesis, besides promoting cell survival^7^. In papillary thyroid cancer cells, TNFα can induce Epithelial–mesenchymal transition (EMT) and thereby promote aggressiveness and metastatic potential^8^. Thus, MADD being an adaptor protein and possessing the ability to activate ERK in TNFα signaling might have a role in cancer metastasis, which needs to be investigated.

Due to its diverse functions in inflammation and apoptosis, therapeutic targeting of TNFα might result in a compromised immune system and severe toxic side-effects. Thus, downstream molecules of TNFα signaling which are cancer-specific might be better therapeutic targets to prevent systemic toxicity. Based on its cancer cell-specific expression and ability to modulate TNFα/ERK axis which can alter both cancer growth and metastatic potential, we hypothesized that MADD could also be a cancer-specific molecular target for ATC therapeutics.

To address this, we first used in vitro and in vivo models to investigate the consequences of MADD knockdown on ATC growth. We next examined the effects of MADD ablation on oncogenic and metastatic features such as cell cycle progression, cellular motility, migration, and invasion; clonogenicity, mitochondrial length, and membrane potential. To determine the molecular basis of these effects, we compared the levels of Wnt signaling effector molecule, β-Catenin and EMT markers in MADD depleted cells with untreated control and scramble siRNA-treated cells. Lastly, we validated the anti-metastatic effect of MADD depletion in an orthotopic thyroid cancer model. Thus, this study demonstrates the role of MADD in ATC metastasis and maps the foundation for its potential therapeutic implications.

## Material and Methods

### Cell lines and transfections

We procured three cell lines (8505C, C643 and HTH7) from University of Colorado Cancer Center, Aurora, CO, USA. All cell lines were authenticated and tested for mycoplasma and other pathogens before experimental initiation (Idexx Laboratories, Inc). Cells were cultured in RPMI medium with 10% fetal bovine serum and 1× antibiotic-antimycotic (Thermo Fisher Scientific) and incubated at 37 °C in a humidified CO_2_ incubator. For all transfections, we used previously-characterized MADD specific siRNA on the basis of its specificity and effectiveness to knockdown MADD, as described before^4,9^. Briefly, the sequences used in this investigation were (MADD siRNA: [(Sense strand: 5′-CGGCGAAUCUAUGACAAUCTT-3′) (Antisense strand: 5′-GAUUGUCAUAGAUUCGCCGTT-3′)] and scramble siRNA: [(Sense strand: 5′-UUGCUAAGCGUCGGUCAAUTT-3′) (Antisense strand: 5′-AUUGACCGACGCUUAGCAATT-3′). We used Lipofectamine RNAiMax solution for transfections as per manufacturer’s recommendations (Thermo Fisher Scientific).

### qRT-PCR

Total pooled RNA from five normal human thyroid tissues was commercially purchased from BioChain Institute, Inc. The sequence of primers used for cDNA synthesis was MADD Forward: 5′-GCCAGCAGCCTCTATCGG-3′, MADD Reverse: 5′-GCCCAAATACTTTCAGAC-3′, GAPDH Forward: 5′-TGTGGGCATCAATGGATTTGG-3′ and GAPDH Reverse: 5′-ACACCATGTATTCCGGGTCAAT-3′. The cDNA was prepared by using iScript cDNA Synthesis Kit (Bio-Rad) and SYBR Green Supermix (Bio-Rad), respectively. Real-time PCR was run by using Bio-Rad CFX Connect Real-time System. Other gene-specific forward and reverse primers used were 5′-CAGGTTGGACAGTTCACAGG-3′ and 5′-ACAGCTGGAGTTGGATGGAC-3′ for *Cyclin D1* and 5′-TGAACACAGCGAATGTTTCC-3′ and 5′-TTAGGAGCGCTCAGGTCTGT-3 for *c-Myc*^10^, 5′-TTGGACTGTCAGGAATGAGG-3′ and 5′-GCAGCACAAAATTCTCCGTG-3′ for MT1-MMP^11^.

### Cell proliferation and Cell cycle analysis

After 48 h of transfection, an equal number of the untreated control, scramble siRNA-transfected, and MADD siRNA-transfected cells were re-plated for the assay. Cells were incubated at 37 °C in a CO_2_ incubator for 24, 48 and 72 h. At every time point, MTT was added to the plate, followed by the incubation for 2 h at 37 °C in a CO_2_ incubator. After DMSO addition and formazan crystal dissolution, absorbance was recorded at 590 nm. Propidium iodide (PI) staining was used for flow-cytometry based cell cycle analysis as described previously^12^. Further, cell cycle halt was confirmed by Western blotting by probing for G_0_-G_1_ (Cdk2 pTyr15) and G_2_-M (Histone H3 pSer10) phase markers using cell cycle marker cocktail (Abcam), as described earlier^13^.

### Colony Formation Assay

Effect of MADD silencing on cellular clonogenicity was determined in both anchorage-dependent as well as anchorage-independent conditions by using 2D clonogenic assay and 3D soft-agar method respectively, as described earlier^14,15^.

### Cellular motility, migration and invasion assay

Wound-closure assay was performed to determine the effect of MADD ablation on cellular motility. We used Boyden chamber (Becton Dickinson Labware, Bedford, MA) for migration and invasion assays as described earlier^16^. For analysis, we determined the percentage of migrated cells by counting a number of cells in the lower chamber and the number of live unmigrated cells (trypan blue negative) in the upper chamber. Percentage invasion was similarly assessed by counting the cells that invaded through Matrigel.

### Mitochondrial length determination

After 48 h of transfection, mitochondria were labeled with MitoTracker Red FM (Thermo Fisher Scientific), fixed with 4% paraformaldehyde (pH 7.4), permeabilized with 0.5% Igepal (Sigma-Aldrich) and blocked, then subjected to Fluorescein Phalloidin (Thermo Fisher Scientific) staining. DAPI (Sigma-Aldrich) was used as a nuclear marker. Different fields were captured by using Keyence inverted fluorescence microscope, and the length of the mitochondria was measured for quantitative purposes using Image pro-plus software, as described earlier^17^.

### Mitochondrial membrane potential

For JC-1 (5,5′,6,6′-tetrachloro-1,1′,3,3′-tetraethyl benzimidazole-carbocyanine) staining, 5 × 10^4^ untreated control, scramble siRNA-transfected and MADD siRNA-transfected cells (post 48 h of transfection) were seeded on to the 24-well plate. After 24 h, the cells were stained with JC-1 solution (10 µM) for 30 min at 37 °C. After two PBS washes, images were captured on the Keyence microscope and processed with BZ-X software.

### Preparation of Nuclear and cytosolic extracts

The nuclear and cytosolic fractions of 8505 C, C643 and HTH7 cells were prepared as described previously^18^. LaminB1 and Tubulin were used as nuclear and cytosolic markers respectively.

### Western blotting

The effect of MADD depletion on EMT programme and Wnt signaling was examined by western blotting. Briefly, 50 μg of whole cell lysates of the untreated control, scramble siRNA-transfected and MADD siRNA-transfected cells were subjected to SDS-PAGE, transferred on to the PVDF membrane, blocked with 5% skimmed non-fat milk and incubated with different primary antibodies at 4 °C overnight on a rocker shaker. All the primary antibodies used are listed in Supplementary Table S1. After four washes with 0.1% TBST_20_, the membrane was incubated with corresponding HRP conjugated secondary antibody [Anti-mouse IgG (Cell Signaling Technology); Anti-rabbit IgG (Cell Signaling Technology)] prepared in blocking solution (5% milk in 0.1% TBST_20_) for an hour at room temperature. After four washes with 0.1% TBST_20_, blots were developed in Kodak X-OMAT 2000A Processor machine after adding Pierce ECL Western Blotting Substrate (ThermoFisher Scientific). Densitometric values were analyzed using Image J software.

### Athymic nude mice, Orthotopic tumor implantation Surgery and siRNA treatment

Athymic nude mice were procured from Charles River Laboratories and were maintained in a pathogen-free facility of the biological resources laboratory (BRL) at the University of Illinois at Chicago (Chicago, IL). All animal experiments were approved by the Animal Care and Use Committee at the University of Illinois at Chicago and Institutional Animal Care and Use Committee (IACUC) at Jesse-Brown VA Medical Center, Chicago, IL. Mice had an *ad-lib* normal diet and were monitored daily for optimal health. 0.5 × 10^6^ 8505 C cells were surgically implanted in the left lobe of the thyroid to generate orthotopic tumors in female athymic nude mice as previously described^19^. After 35 days, mice were randomized into three treatment arms: untreated control, scramble siRNA and MADD siRNA (*N* = 5 per group). Treatment regimen required a total of five intra-tumoral doses of 20 nmoles MADD siRNA or scramble siRNA in invivofectamine reagent (Thermofisher Scientific) administered on every alternate day. Tumor dimensions were measured daily for volumetric calculations with the formula: *V* = 1/2 × l × b^2^, where *l* is longest diameter and *b* is perpendicular breadth. On day 46, mice were sacrificed, and tumor and lung tissue were collected. Samples were fixed, sectioned and subjected to Hematoxylin and Eosin (H&E) staining and Immunohistochemistry (IHC).

### Immunohistochemical (IHC) studies and quantification

IHC studies were performed on the mouse tumor tissue sections as earlier described^16^. Diaminobenzidine and hematoxylin were used as a substrate and nucleus counter-stain, respectively. Slides were mounted using Acrymount (StatLab) and microphotographed in the phase contrast/brightfield settings using Keyence microscope.

### Statistical analysis

All data are represented as mean ± s.d. and differences between groups were evaluated with a two-tailed student *t*-test. Statistical analysis was performed in the GraphPad Prism 7, and a *P* < 0.05 was considered statistically significant.

## Results

### MADD depletion reduces ATC growth in vitro and in vivo

**Fig. 1:**
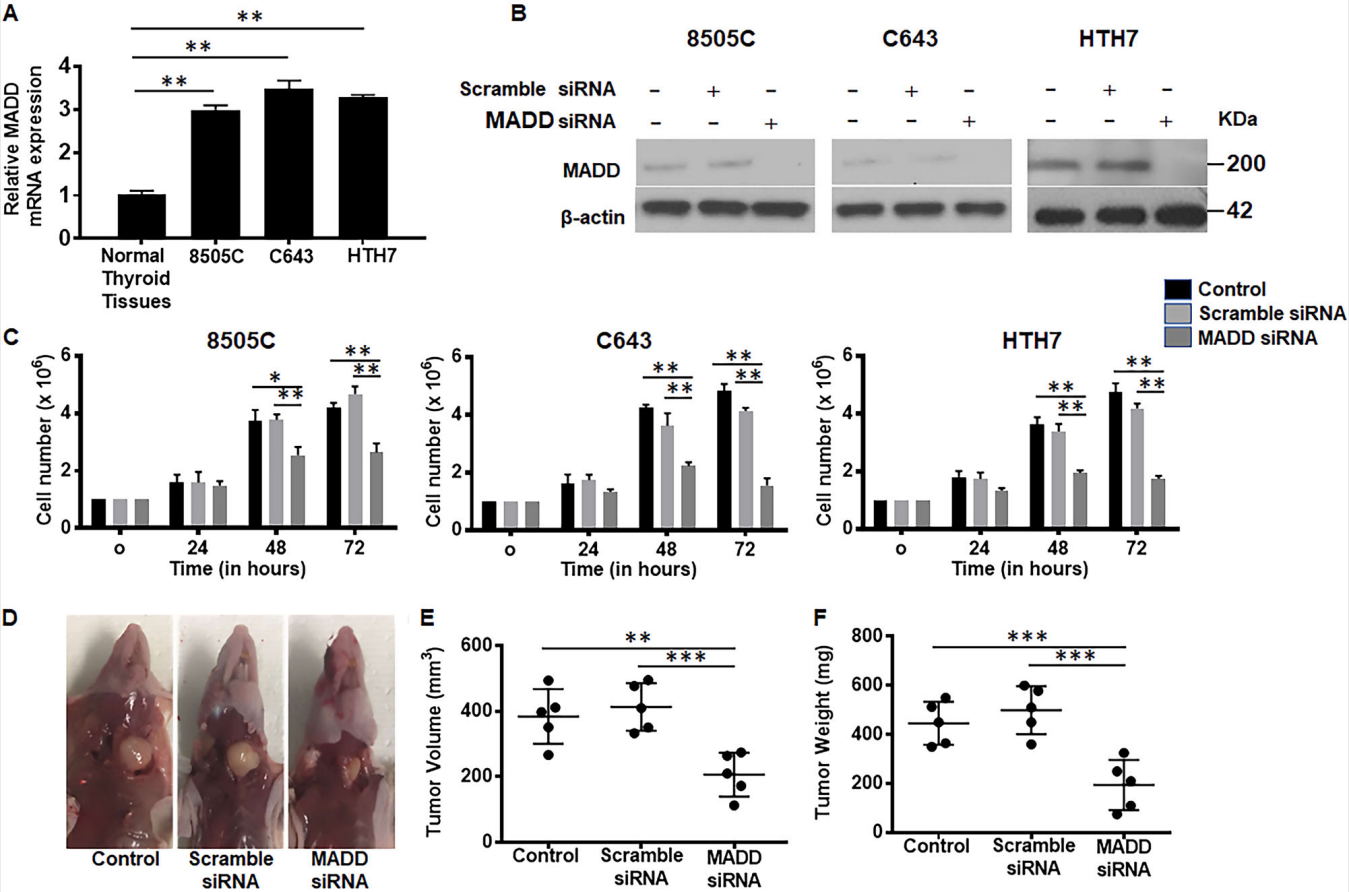
*MADD* gene silencing reduces ATC growth in vitro and in vivo: **a** Quantitative RT-PCR showed significantly higher expression of MADD mRNA in 8505 C, C643 and HTH7 cells in comparison to the normal human thyroid tissues. **b** MADD siRNA treatment resulted in a significant reduction in MADD protein expression, depicted by Western blotting. β-actin was used to normalize the expression levels. **c** MTT assay exhibited a significant reduction in proliferation MADD depleted cells after 72 h in 8505 C, C643 and HTH7 cells. **d** Representative photograph of 8505 C orthotopic tumor-bearing mice treated with MADD siRNA in comparison to the untreated control and scramble siRNA-treated mice. **e**, **f** Intra-tumoral treatment with MADD siRNA showed a significant reduction in the tumor volume and weight, as compared to the untreated control and scramble siRNA-treated tumors. All the values represent the mean ± SD of triplicate samples from a typical experiment. *P < 0.05, **P < 0.005, and ***P < 0.0005, by the two-tailed student’s *t* test

*qRT-PCR* showed that MADD overexpression was consistent in all ATC cell lines irrespective of their genetic background and mutational profiles (*P* < 0.005; Fig. 1a) (Supplementary Table S2). Western blotting showed that MADD siRNA effectively depleted MADD at the protein level after 48 h of transfection in all cell lines (Fig. 1b). Densitometric analysis showed that MADD siRNA transfection resulted in 71.8 ± 0.059%, 69.2 ± 0.07% and 70.2 ± 0.04% reduction in MADD protein levels in 8505 C, C643 and HTH7 cells respectively, as compared to scramble siRNA-transfected cells (*P* < 0.0005) (Supplementary Figure S1A). First, we investigated the effect of MADD ablation on cell proliferation in a three-day course and found that MADD siRNA -transfected cells exhibited cellular growth stagnation whereas control and scramble siRNA-transfected cells continued to grow normally, post 48 and 72 h of transfection (*P* < 0.005) (Fig. 1c). Second, we examined the therapeutic implications of MADD targeting in an orthotopic ATC model in athymic nude mice. In vivo, MADD siRNA-treated tumors (118.4 ± 43.18 mm^3^) had significantly reduced tumor volume as compared to untreated control (226.2 ± 79.62 mm^3^; *P* < 0.005) and scramble siRNA-treated tumors (254 ± 38.18 mm^3^; *P* = 0.0005) (Fig. 1d, e). Concurrently, we noted a significant reduction in tumor weight of MADD siRNA-treated (194 ± 102.2 mg) in contrast to untreated control (445.6 ± 88.17 mg; *P* < 0.0005) and scramble siRNA-treated tumors (499.6 ± 97.83; *P* < 0.0005) (Fig. 1f). These in vivo findings recapitulated the inhibitory effects of MADD knockdown on ATC growth indicating its potential as a therapeutic target.

### MADD abrogation induces cell cycle arrest and compromises clonogenicity

**Fig. 2:**
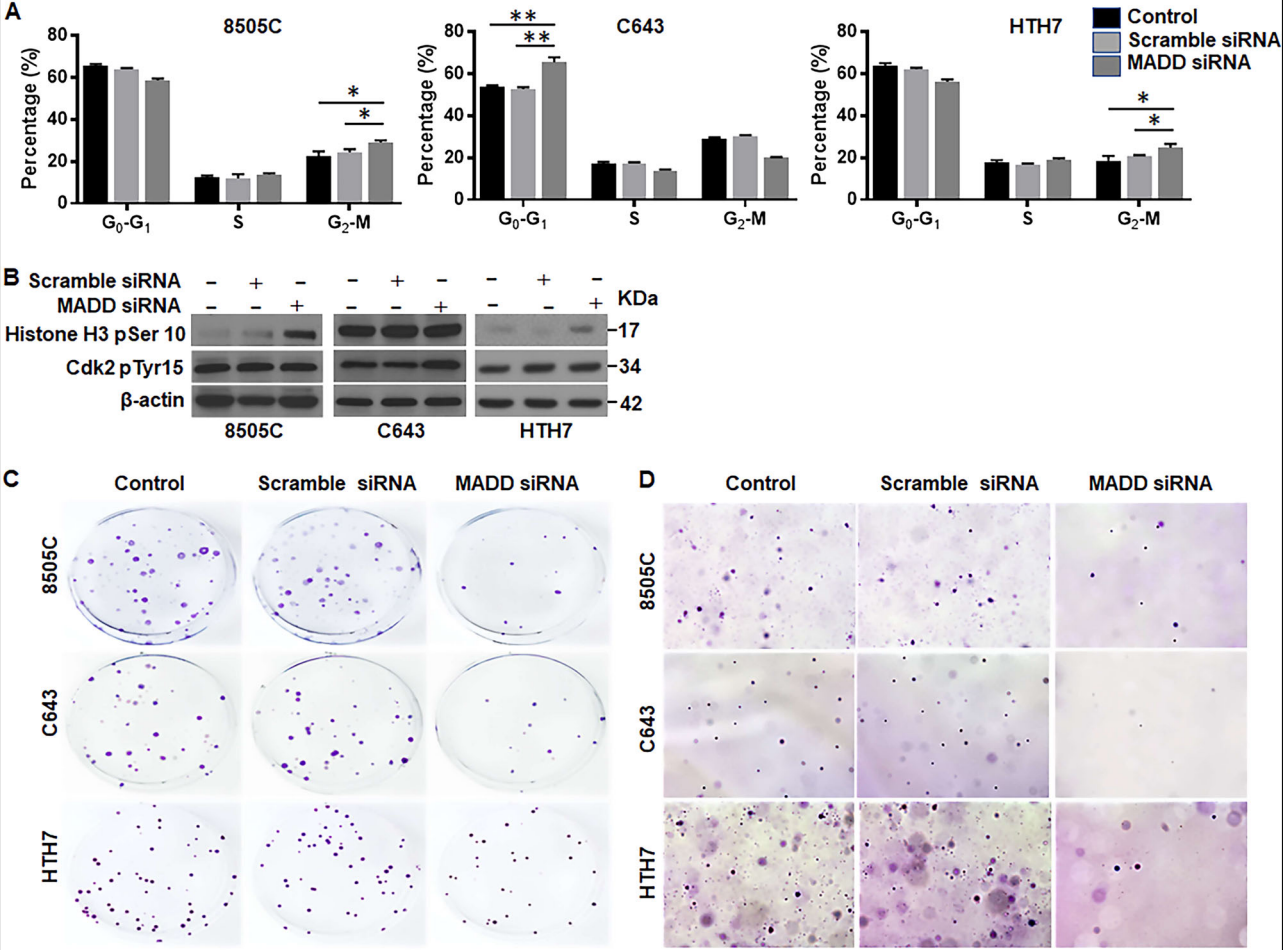
MADD depletion halts cell cycle and suppresses clonogenic ability of ATC cells: **a** PI staining-based flow-cytometry revealed cell cycle arrest at a G_2_-M phase in 8505 C and HTH7 whereas C643 exhibited G_0_-G_1_ arrest. **b** Western blotting analysis of G_0_–G_1_ (Cdk2 pTyr15) and G_2_-M arrest (Histone H3 pSer10) markers confirmed cell cycle halt at different phases in ATC cells. **c** Representative photographs showing the lower number of colonies formed in MADD depleted cells as compared to the untreated control and scramble siRNA-transfected cells in all ATC cell lines in 2D colony forming assay. **d** Representative microphotographs showing the effect of MADD siRNA on growth in 3D soft agar assay. Similar results were obtained in two additional independent experiments

To investigate whether the anti-proliferative effects of MADD siRNA involved cell cycle arrest, we employed PI staining to determine the effect of MADD knockdown on cell cycle progression. Our data revealed cell cycle arrest at different phases in MADD depleted 8505 C, C643 and HTH7 cells. In particular, 8505 C cells showed a modest G_2_-M arrest upon MADD ablation (27.75 ± 1.48%) in contrast to the untreated control (22.54 ± 2.14%; *P* < 0.05) and scramble siRNA-transfected cells (24.28 ± 1.51%; *P* < 0.05). Likewise, HTH7 cells also exhibited cell cycle arrest at a G_2_-M phase upon MADD siRNA treatment (24.78 ± 1.67%) as compared to the untreated control (18.57 ± 2.27%; *P* < 0.05) and scramble siRNA-transfected cells (21.47 ± 0.83%; *P* < 0.05) (Fig. 2a). In contrast, C643 cells demonstrated cell cycle arrest at a G_0_-G_1_ phase upon MADD ablation (65.4 ± 2.34%) in comparison to the untreated control (53.83 ± 0.55%; *P* < 0.005) and scramble siRNA-transfected cells (52.57 ± 0.89%; *P* < 0.005) (Fig. 2a). To further confirm this phenotype, we probed the cells with Cdk2 pTyr15 (G_0_-G_1_ arrest marker_)_ and Histone H3 pSer10 (G_2_-M arrest marker). Consistent with our PI staining results, 8505 C and HTH7 cells (but not C643 cells) exhibited elevated levels of Histone H3 pSer10 levels upon MADD silencing, as compared to control and scramble siRNA-transfected cells, suggesting that MADD knockdown was associated with G2-M phase halt in 8505 C and HTH7 cells. Whereas MADD depletion in C643 resulted in increased expression of Cdk2 pTyr15, in comparison to control and scramble siRNA-transfected cells, indicating G_0_–G_1_ stalling in C643 cells (Fig. 2b). Thus, MADD abrogation can induce cell cycle arrest in ATC cells at different phases.

Next, we determined whether MADD depletion can inhibit the clonogenic potential of ATC cells in anchorage-dependent and anchorage-independent conditions. Both colony forming (2D) and soft-agar assays showed that MADD siRNA-transfected ATC cells exhibited significantly reduced colony forming potential in comparison to the untreated control and scramble siRNA-transfected 8505C, C643 and HTH7 cells (Fig. 2c, d). Quantitative assessment of colonies revealed that MADD abrogation significantly reduced the growth of ATC cells in both anchorage-dependent as well as anchorage-independent manner (*P* < 0.005) (Supplementary Figures S1B and S1C).

### MADD knockdown blocks cellular motility, migration, and invasion

**Fig. 3:**
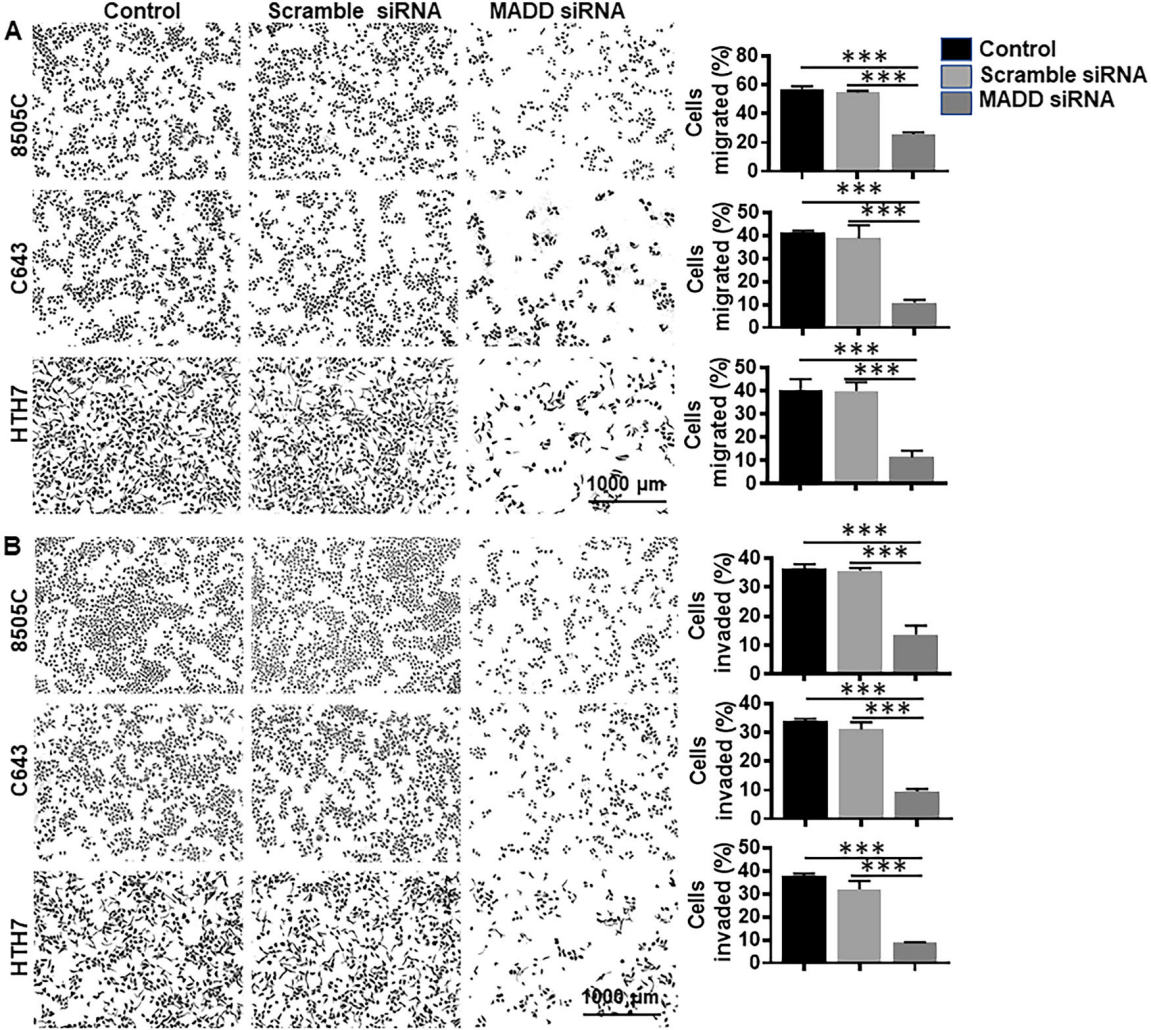
MADD gene silencing reduces migration and invasion in ATC cells: **a** Transwell membrane insert assay showed inhibition of migratory potential in MADD siRNA-transfected cells as compared to untreated control and scramble siRNA-transfected cells. **b** All three cell lines exhibited a significant reduction in invasive ability upon MADD knockdown. Values show mean ± SD, *P < 0.05, **P < 0.005, and ***P < 0.0005, by two-tailed Student’s *t* test

Cellular growth inhibition by MADD silencing in soft agar plates prompted us to examine the role of MADD in other malignant properties of ATC cells. First, our wound-healing experiment revealed a significant reduction in the ability of MADD siRNA-transfected cells to migrate (fill the gap) as compared to the untreated control and scramble siRNA-transfected cells (Supplementary Figures S2–S4). Even after 24 h, MADD ablated 8505 C cells showed no or minimal reduction in the wound width (2.3 ± 0.83%), MADD siRNA-transfected C643 cells exhibited 43.4 ± 2.3% reduction in the gap, and MADD abrogated HTH7 cells demonstrated 16.6 ± 2.1% reduction in the scratch width, as compared to control and scramble siRNA-transfected cells, suggesting that MADD depletion significantly compromisedcellular motility of these ATC cells (*P* < 0.0005; *P* < 0.0005; *P* < 0.0005) (Supplementary Figures S2–S4). Second, data from Boyden chamber based transwell-insert assay showed a significant reduction in the percentage of migrated cells in the lower chamber upon MADD ablation in 8505 C (*P* < 0.0005), C643 (*P* < 0.0005) and HTH7 cells *(P* < 0.0005) (Fig. 3a). A similar trend was observed in percentages of invaded cells upon MADD silencing in all three cell lines (*P* < 0.0005) (Fig. 3b). In total, MADD knockdown significantly suppressed cellular motility, migratory and invasive potential of ATC cells in vitro.

### MADD gene silencing causes a reduction in mitochondrial length and mitochondrial potential

**Fig. 4:**
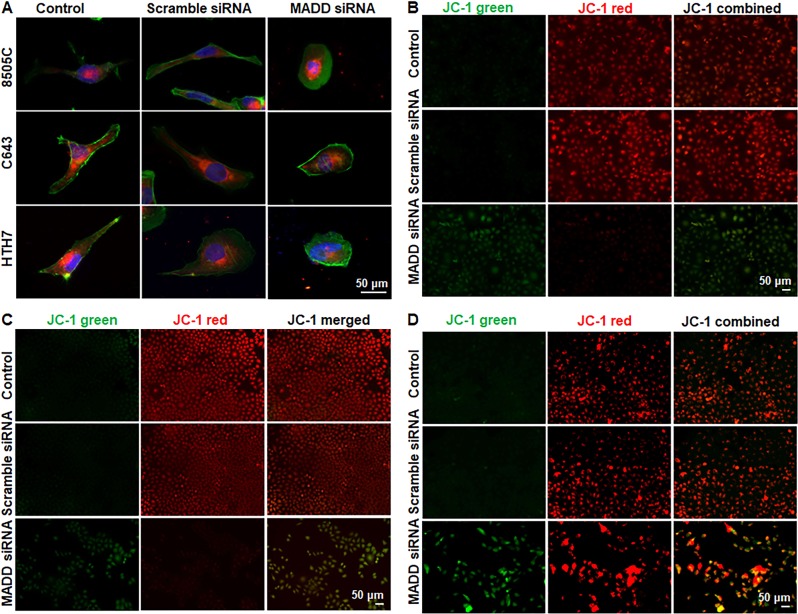
Effect of MADD abrogation on mitochondrial length and membrane potential: **a** Representative microscopic images depicting reduced mitochondrial length in MADD ablated cells versus untreated control and scramble siRNA-treated cells. Mitochondria are stained in red (Mitotracker RedFM), F-actin is stained in green (phalloidin) and nuclei are stained blue (DAPI). **b**–**d** Representative microscopic images showing a reduction in mitochondrial membrane potential in (**b**) 8505 C, (**c**) C643 and (**d**) HTH7 cells. In metabolically active cells with polarized/hyperpolarized mitochondria, JC-1 is concentrated inside mitochondria and forms J aggregates (red-colored), which has a 590 nm emission. When there is mitochondrial depolarization, JC-1 cannot form aggregates and exists as a green fluorescent monomer (emission at 530 nm)

As cellular migration and invasion are high energy driven processes and require high mitochondrial activity^20^, we investigated whether MADD silencing impacts mitochondrial length or membrane potential. Our data revealed that MADD down-regulation is associated with reduced mitochondrial length in ATC cells (Fig. 4a). Quantitative assessment depicted that MADD siRNA-transfected ATC cells exhibited a significant percentage reduction in mitochondrial length as compared to the untreated control and scramble siRNA-transfected 8505C (*P* < 0.005), C643 (*P* < 0.005) and HTH7 (*P* < 0.005) cell lines (Supplementary Figure S1D). Since alteration in cellular morphology induced by apoptosis and autophagy can also cause shrinkage of mitochondrial length, we wanted to establish that alterations in mitochondrial length were due to mitochondrial stress, and not apoptosis. We used an alternative method of determining mitochondrial health (JC-1 staining) which suggested a significant reduction in mitochondrial membrane potential upon MADD abrogation in all three ATC cells, as depicted by increased green color JC1 monomers in MADD depleted cells (Fig. 4b–d). In conclusion, MADD depletion correlates with reduced mitochondrial length and mitochondrial membrane potential indicating the possibility of a mitochondrial collapse in MADD depleted cells.

### MADD siRNA-induced-reduction in migratory/invasive potential is concomitant with reduced EMT programme

**Fig. 5:**
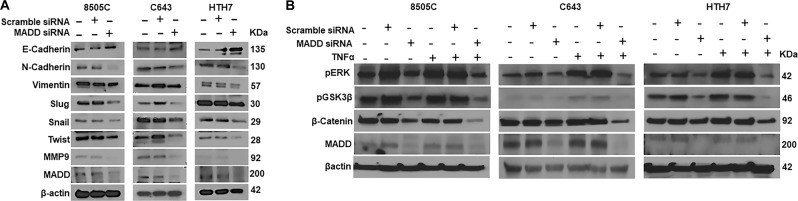
Effect of MADD depletion on EMT program and Wnt Signaling: **a** Western blotting of MADD depleted cells, as compared to the untreated control and scramble siRNA-treated cells, showed a significant reduction in N-Cadherin, Vimentin, Snail, Slug, Twist, and MMP9; and an increase in E-Cadherin expression. **b** TNFα addition resulted in an increase of pERK, pGSK3β and β-catenin levels, prominently in C643 and hTh7 cells while 8505C cells demonstrated a moderate response to TNFα addition. MADD siRNA inhibited TNFα-induced Wnt signaling in ATC cells, as depicted by reduced levels of pERK, pGSK3β and β-catenin in all cells. MADD siRNA, by itself, did not alter β-catenin expression levels in C643 and HTH7 cells

EMT precedes cancer cell migration and invasion. Therefore, we determined the levels of EMT markers in ATC cells upon MADD knockdown by Western blotting which showed a significant reduction in metastasis promoting matrix metalloprotease, MMP9, in MADD siRNA-transfected cells in comparison to the untreated control and scramble siRNA-transfected 8505C (73.3%), C643 (59.1%) and HTH7 (76.6%) cells. Our data also demonstrated a significant reduction of transcription factors such as Slug (8505C: 39.2%; C643: 45.3%; HTH7: 21.5%), Snail (8505C: 44%; C643: 12.55%; HTH7: 28.08%) and Twist (8505C: 27%, C643: 50.7%; HTH7:63.2%) and mesenchymal phenotype markers such as N-Cadherin (8505 C: 51.36%; C643: 54.23%; HTH7: 51.1%) and Vimentin (8505C: 62.1%; C643: 18.41%; HTH7: 39.5%), respectively (Fig. 5a). E-Cadherin (Epithelial cell adhesion molecule) expression was increased in MADD siRNA-transfected cells which indicated that MADD depletion restored their epithelial phenotype in 8505C (16.3%), C643 (16.6%) and HTH7 (175.3%) cell lines (Fig. 5a). Cumulatively, our data confirmed that MADD depletion resulted in inhibition of EMT programme in ATC cells.

### MADD siRNA inhibits EMT by blocking β-Catenin through TNFα/ERK/GSK3β axis

EMT activation is driven by several factors such as hypoxia, TGFβ, Notch and Wnt^21^. However, the classical and canonical pathway responsible for EMT activation in cancer is Wnt Signaling^22,23^. It is associated with aggressiveness, metastatic potential, and stemness in several cancer entities including thyroid cancer^24–27^. The aberrant nuclear activity of β-Catenin has been reported in ATC tissue specimens and is known to induce EMT^24,26,28–31^. To further validate the role of β-Catenin in EMT in ATC cells, we inhibited β-Catenin nuclear activity by using an inhibitor PRI-724 and found a significant reduction in N-cadherin and increased levels of E-Cadherin in all ATC cells (Supplementary Figure S5A). This suggested that β-Catenin is at least in part, involved in the activation of EMT in ATC cells.

**Fig. 6:**
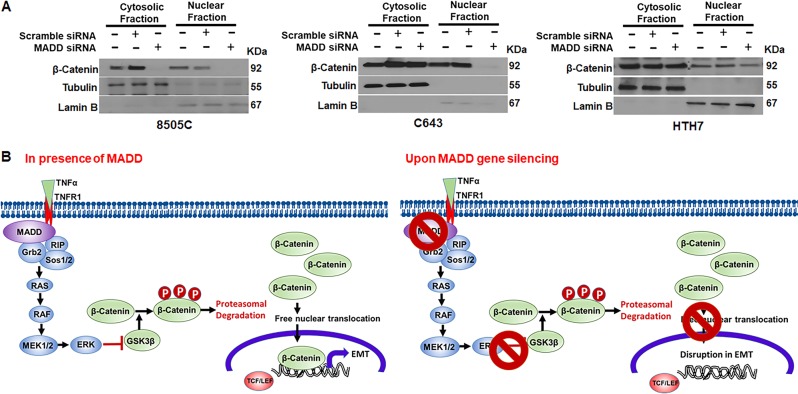
MADD siRNA inhibits β-catenin nuclear translocation in ATC cells. **a** Comparison of cytosolic and nuclear extracts revealed that MADD ablation blocks nuclear translocation of β-catenin in C643 and HTH7 cells. Tubulin and Lamin B were used as the cytosolic marker and nuclear marker, respectively. **b** In summary, In TNFα signaling, MADD recruits at the TNFR1 alongside Grb2 and Sos1/2 and results in the activation of ERK through RAS/RAF/MEK1/2 axis. pERK can phosphorylate GSK3β and render it inactive. GSK3β promotes ubiquitin-based proteasomal degradation of β-catenin by phosphorylating it. Inhibition of GSKβ by pERK can render β-catenin free for nuclear translocation and thereby, promote activation of genes involved in proliferation, de-differentiation, and EMT by displacing the repressor TCF/LEF. Upon MADD depletion by siRNA, ERK is not activated, and consequently, GSK3β is free to phosphorylate and promote β-catenin degradation which eventually results in the inhibition of transcription of β-catenin target genes and consequently, EMT

As a next step, we sought to determine whether MADD siRNA inhibits EMT by modulating Wnt signaling. Our previous studies showed that MADD functions as an adaptor protein and can modulate TNFα/MAPK/ERK signaling^6^. Activation of ERK leading to β-Catenin nuclear activity has been recently shown in cancer^32^. pERK can independently inhibit GSK3β which phosphorylates β-catenin and marks it for ubiquitin-based proteasomal degradation. Inhibitory phosphorylation of GSK3β by pERK can prevent β-Catenin degradation and allow it to freely translocate into the nucleus^24,32^. To test whether MADD siRNA can inhibit β-Catenin nuclear translocation by altering pERK and pGSK3 levels, we compared the levels of pGSK3β, pERK, and β-catenin in MADD siRNA-transfected cells with untreated control and scramble siRNA-transfected cells, with and without TNFα treatment. As depicted in the Fig. 5b, TNFα addition effectively induced an increase in constitutive expression levels of pERK, pGSK3β and β-catenin, predominantly in C643 and HTH7 cells while 8505 C exhibited a moderate increase in pERK, pGSK3β and β-catenin levels. Combination of MADD siRNA and TNFα resulted in substantial reduction of pERK, pGSK3β, β-catenin in all ATC cells. However, MADD siRNA, by itself, had varying effects on β-catenin levels in ATC cells. Upon MADD knockdown, 8505C cells showed a significant decrease in constitutive β-catenin expression levels whereas C643 and HTH7 cells did not. (Fig. 5b). Since β-catenin expression levels were unaltered in C643 and HTh7 cells upon MADD abrogation, we speculated that MADD siRNA might have modulated the functionality of β-catenin by affecting its nuclear translocation. To examine whether MADD siRNA effected β-catenin nuclear translocation, we compared its levels in cytosolic and nuclear extracts. In these two cell lines, the nuclear/cytosolic ratio of β-catenin was significantly reduced [C643 (93.1%) and HTH7 (66.4%)] as compared to the control untreated and scramble siRNA transfected cells (Fig. 6a). Consistent with our previous results (Fig. 5b), we observed that MADD siRNA treatment caused a significant reduction in both cytosolic as well as nuclear β-catenin levels in 8505C cells, suggesting that MADD siRNA could directly or indirectly regulate the expression levels of β-catenin in these cells (Fig. 6a). Collectively, these findings indicated that MADD targeting could inhibit EMT by reducing β-catenin nuclear translocation.

In summary, activation of Wnt signaling results in a higher nuclear/cytosolic ratio of the effector protein, β-catenin, which can activate the gene expression of several transcription factors involved in EMT. In an “off” state, β-catenin exists in an inactive form, bound to the inhibitory complex (consisting of GSK3β and Axin) in the cytoplasm. When the signaling is turned “on”, this complex dissociates itself and allows free translocation of β-catenin into the nucleus. In the nucleus, β-catenin can interact with TCF/LEF and promote the transcription of several genes involved in cell proliferation, de-differentiation and EMT^33^. GSK3β plays a regulatory role as it can phosphorylate β-catenin, marks it for ubiquitin-mediated degradation and hence, prevents its nuclear translocation. TNFα signaling can crosstalk through Wnt signaling *via* pERK and pGSK3β. TNFα-induced pERK can associate with GSK3β and prime its inactivation which leads to β-catenin stabilization and nuclear translocation. siRNA mediated MADD depletion prevents pERK activation and therefore, pGSK3β can perform its function of marking β-catenin ubiquitination which inhibits its nuclear activity and consequently, EMT programme (Fig. 6b). In total, these results provide the mechanistic insights that MADD knockdown might block β-Catenin activity in ATC via inhibiting TNFα/ERK/GSK3β axis.

To validate the loss of functionality of β-catenin upon MADD knockdown, we examined the levels of expression of select β-catenin-target genes, *Cyclin D1*, *Myc* and *MT1-MMP (matrix metalloproteinase)* that are relevant in thyroid cancer^27^. Our qRT-PCR data showed that MADD gene silencing is associated with a significant reduction in *Myc* and *MT1-MMP* mRNA expression in all ATC cells, suggesting that MADD siRNA inhibits, at least in part, β-catenin transcriptional activity (Supplementary Figure S5B). We did not observe a similar trend in *Cyclin D* mRNA levels except in C643 cells, possibly due to its transcriptional regulation by factors other than β-catenin in these cells.

### MADD siRNA inhibits ATC metastasis in vivo

**Fig. 7:**
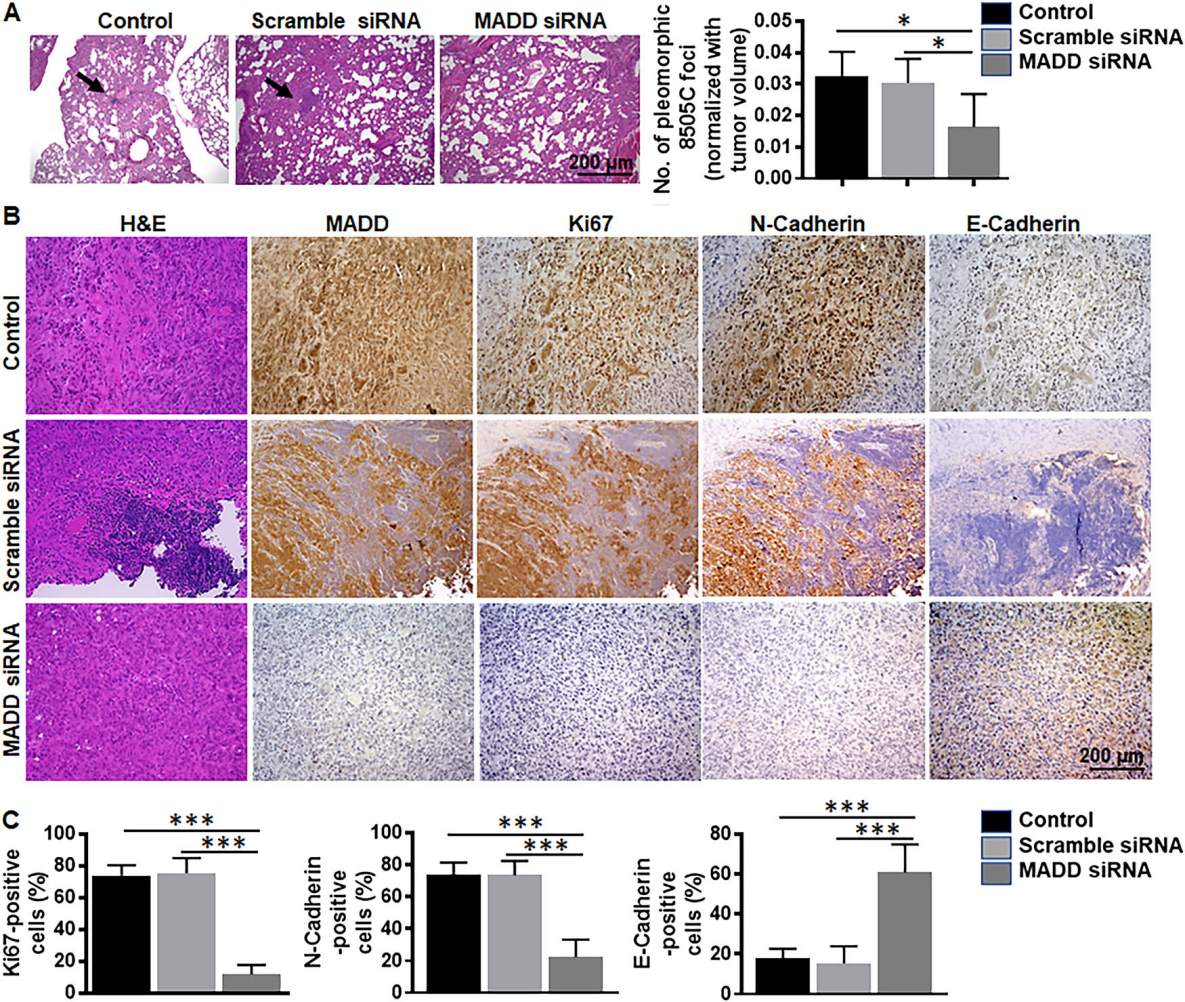
MADD knockdown inhibits ATC metastasis in vivo. **a** Representative H&E stained lung tissue sections showing pleomorphic 8505 C foci in control and scramble siRNA-treated mice but not in MADD siRNA-treated mice. Protein quantitative assessment shows the significant reduction in the ratio of a number of pleomorphic 8505C foci/tumor volume in MADD siRNA-treated mice, as compared to the untreated control and scramble siRNA-treated mice (*N* = 5). **b** Representative specimens of tumor tissues from each treatment group showing H&E staining, MADD, Ki67, N-Cadherin and E-Cadherin expression (left to right). MADD siRNA treatment caused significant reduction of MADD protein in the tumor tissue in vivo. **c** MADD siRNA-treated tumors revealed a substantial decrease in Ki67 and N-Cadherin expression levels. In contrast, E-Cadherin expression was increased in MADD siRNA-treated tumors, suggesting MADD depletion results in restoration of epithelial phenotype in vivo

We further validated the phenotype by performing histopathological analysis of lung tissues (most common metastatic site) of orthotopic ATC bearing mice. H&E staining and pathological assessment revealed a reduced number of pleomorphic 8505 C foci in MADD siRNA-treated mice (3.8 ± 2588) versus untreated control (12.6 ± 4.615; *P* < 0.05) and scramble siRNA-treated mice (12.2 ± 3.782; *P* < 0.05) (Fig. 7a). Immunohistochemical analysis of cell proliferation (Ki67) and EMT (N-Cadherin and E-Cadherin) markers showed that in MADD siRNA-treated tumors, had a significant reduction of Ki67 expression and N-Cadherin as compared to the untreated control and scramble siRNA-treated tumors (*P* < 0.0005) (Fig. 7b, c). In contrast, E-Cadherin was enhanced in MADD siRNA-treated tumors (*P* < 0.0005) (Fig. 7b, c), suggesting that MADD siRNA can reduce ATC metastasis in vivo.

## Discussion

ATC has dismal prognosis due to its undifferentiated and aggressive phenotype^34^. Even radio-iodine based therapy does not kill these aggressive tumors as they lack competent sodium-iodide symporters to absorb iodine unlike differentiated thyroid cancers (DTCs)^35^. With the majority of the ATC patients inevitably presenting with metastasis at the time of diagnosis, the overall survival is ~ six months^35^. Clearly, new therapeutic strategies are desperately needed.

Our previous studies showed that MADD is necessary and sufficient for cancer cell survival^4^. MADD is overexpressed in different cancers including thyroid cancer^36^, breast cancer^37^ and ovarian cancer^38^ and its knockdown causes spontaneous apoptosis^4,5,39^. Consistent with our previous findings, this study demonstrated that MADD knockdown suppressed proliferative capacity in ATC cells, both in vitro and in vivo. 8505 C exhibited a lower degree of MADD siRNA-induced anti-proliferative effects, in comparison to C643 and HTH7. It is interesting to note that 8505 C possesses BRAFV600E mutation whereas other cell lines do not (Supplementary Table S2). All three cell lines have Ras mutations, but due to an additional BRAFV600E mutation in 8505 C, this cell line might have other hyperactive signaling pathways contributing to cellular proliferation despite MADD depletion. This warrants further investigation to determine how BRAFV600E mutation affects MADD siRNA-induced inhibition of cellular proliferation.

A major challenge in ATC management is its aggressiveness and high metastatic potential. In this study, we found that MADD abrogation correlated with suppressed cellular motility, migratory and invasive potential of ATC cells in vitro. Recent studies have shown that mitochondrial dysfunction can lead to compromised cellular migration and invasion in cancer^17,40^. Active/healthy mitochondria are usually elongated and have polarized membrane potential. Our data showed a sharp decrease in mitochondrial length as well as depolarization upon MADD knockdown in ATC cells (Fig. 4). Thus, it is quite possible that MADD siRNA-induced anti-migratory/invasive effects, at least in part, is due to perturbations in mitochondrial function. The exact mechanism of mitochondrial involvement in MADD siRNA-induced inhibition of migration/invasion warrants further studies.

Cancer cell migration and Invasion comprises EMT activation which epitomizes the metastatic process in cancer. It results in trans-differentiation of epithelial cells into motile, invasive mesenchymal cells, which can disseminate and form a secondary tumor. Interestingly, MADD silencing resulted in EMT inhibition in all ATC cells tested (Fig. 5a). To decipher the possible molecular mechanism of MADD siRNA mediated EMT inhibition, we screened out the possible signaling cascades which can induce EMT and are specific to ATC development. In this context, Wnt signaling imparts dedifferentiation phenotype and metastatic features to ATC and is hyperactivated in ATC as compared to normal thyroid tissues and DTCs^24,25,29^. The downstream effector of Wnt signaling, β-catenin can regulate the expression of several EMT-related transcription factors such as *Twist*, *Slug* and *Snail2*^41^. The important role of β-catenin gene (*CTNNB1*) in thyroid cancer pathogenesis has been established by several studies^24,27,28,32^. Also, *CTNNB1* (*β-catenin*) is frequently mutated and is constitutively active in poorly differentiated thyroid cancers and ATC^29,42–44^. We also confirmed that β-catenin is at least in part responsible for EMT activation in ATC cells (Supplementary Figure S5).

The possible connection of MADD to β-Catenin regulation stemmed from our previous studies wherein, we showed that MADD protects cells from TNFα mediated cytotoxicity in cancer cells by facilitating TNFα induced MAPK activation^5^. However, the contribution of MADD in other TNFα functions such as migration, invasion, and angiogenesis remained elusive^45^. Recently, it is shown that pERK can promote β-catenin activity by blocking its regulatory molecule GSK3β. This prevents the ubiquitination of β-catenin, keeping it in an active form^32^. As illustrated in Fig. 6b, our data showed that MADD siRNA can significantly inhibit the TNFα mediated activation β-Catenin likely by targeting TNFα/ERK/GSK3β axis and thereby, blocking EMT activation. Furthermore, intra-tumoral administration of MADD siRNA resulted in reduced number of pleomorphic foci in the treated mouse lung tissue and reduced N-Cadherin expression in tumor tissues. Hence, this investigation provides significant evidence to establish a role for MADD in cancer metastasis.

ATC metastasis is not extensively studied, perhaps due to the rarity of the disease. Moreover, there are very few molecules which exhibited a correlation with migration and invasion in ATC. In this regard, Laminin-5γ-2 (LAMC2) showed a correlation with ATC growth, migration and invasion by modulating EGFR signaling^46^. Similarly, inhibition of histone deacetylase exhibited suppression of migration and invasion of ATC cells in vitro^47^. However, none of these studies validated the role of these molecules in metastasis in vivo. The orthotopic thyroid cancer tumor model is an established system to investigate the pre-clinical potential of molecules and provided the opportunity to evaluate the effect of MADD knockdown in a pertinent in vivo system where tumor architecture is preserved^19^. The primary clinical challenge with MADD siRNA treatment would be its tumor-specific delivery at an optimal concentration. To address this, future studies are required to identify a MADD specific small molecule inhibitor or antagonist which can modulate MADD expression levels in a tumor-specific manner. ATC is a highly aggressive cancer with a very high degree of therapeutic resistance. However, our mechanistic studies revealed several nodal points which can be targeted, in combination with MADD knockdown, to prevent/reduce metastasis. In conclusion, this study paves the way to explore potential clinical utility of MADD knockdown for treating ATC.

## Supplementary information

Supplementary FIgure S1

Supplementary FIgure S2

Supplementary FIgure S3

Supplementary FIgure S4

Supplementary FIgure S5

supplemental figure legends

## References

[1] Siegel RL, Miller KD, Jemal A (2018). Cancer statistics, 2018. CA Cancer J. Clin..

[2] Liu TR (2016). Treatment and prognosis of anaplastic thyroid carcinoma: a clinical study of 50 cases. PLoS ONE.

[3] Tiedje, V. et al. Anaplastic thyroid carcinoma: review of treatment protocols. *Endocr. Relat. Cancer*10.1530/erc-17-0435 (2018). 10.1530/ERC-17-0435 29295821

[4] Mulherkar N, Ramaswamy M, Mordi DC, Prabhakar BS (2006). MADD//DENN splice variant of the IG20 gene is necessary and sufficient for cancer cell survival. Oncogene.

[5] Kurada BR (2009). MADD, a splice variant of IG20, is indispensable for MAPK activation and protection against apoptosis upon tumor necrosis factor-alpha treatment. J. Biol. Chem..

[6] Al-Zoubi AM (2001). Contrasting effects of IG20 and its splice isoforms, MADD and DENN-SV, on tumor necrosis factor alpha-induced apoptosis and activation of caspase-8 and -3. J. Biol. Chem..

[7] Wu Y, Zhou BP (2010). TNF-α/NF-κB/Snail pathway in cancer cell migration and invasion. Br. J. Cancer.

[8] Lv N (2015). Inflammatory mediators, tumor necrosis factor-α and interferon-γ, induce EMT in human PTC cell lines. Oncol. Lett..

[9] Mulherkar N, Prasad KV, Prabhakar BS (2007). MADD/DENN splice variant of the IG20 gene is a negative regulator of caspase-8 activation. Knockdown enhances TRAIL-induced apoptosis of cancer cells. J. Biol. Chem..

[10] Kang YJ (2012). Wnt/β-catenin signaling mediates the antitumor activity of magnolol in colorectal cancer cells. Mol. Pharmacol..

[11] Egawa N (2006). Membrane type 1 matrix metalloproteinase (MT1-MMP/MMP-14) cleaves and releases a 22-kDa extracellular matrix metalloproteinase inducer (EMMPRIN) fragment from tumor cells. J. Biol. Chem..

[12] Kanojia D (2013). Sperm associated antigen 9 plays an important role in bladder transitional cell carcinoma. PLoS ONE.

[13] Zwergel C (2017). Novel coumarin- and quinolinone-based polycycles as cell division cycle 25-A and -C phosphatases inhibitors induce proliferation arrest and apoptosis in cancer cells. Eur. J. Med. Chem..

[14] Franken NAP, Rodermond HM, Stap J, Haveman J, van Bree C (2006). Clonogenic assay of cells in vitro. Nat. Protoc..

[15] Horibata, S., Vo, T. V., Subramanian, V., Thompson, P. R. & Coonrod, S. A. Utilization of the soft agar colony formation assay to identify inhibitors of tumorigenicity in breast cancer cells. *J. Vis. Exp.* 52727, 10.3791/52727 (2015). 10.3791/52727 PMC4542786 26067809

[16] Saini S (2013). Gene silencing of A-kinase anchor protein 4 inhibits cervical cancer growth in vitro and in vivo. Cancer Gene Ther..

[17] Zhao J (2013). Mitochondrial dynamics regulates migration and invasion of breast cancer cells. Oncogene.

[18] Singhirunnusorn P, Suzuki S, Kawasaki N, Saiki I, Sakurai H (2005). Critical roles of threonine 187 phosphorylation in cellular stress-induced rapid and transient activation of transforming growth factor-beta-activated kinase 1 (TAK1) in a signaling complex containing TAK1-binding protein TAB1 and TAB2. J. Biol. Chem..

[19] Nucera C (2009). A novel orthotopic mouse model of human anaplastic thyroid carcinoma. Thyroid.

[20] Desai SalilP, Bhatia, Sangeeta N, Toner M, Irimia D (2013). Mitochondrial localization and the persistent migration of epithelial cancer cells. Biophys. J..

[21] Liao TT, Yang MH (2017). Revisiting epithelial-mesenchymal transition in cancer metastasis: the connection between epithelial plasticity and stemness. Mol. Oncol..

[22] Zhan T, Rindtorff N, Boutros M (2016). Wnt signaling in cancer. Oncogene.

[23] Yao D, Dai C, Peng S (2011). Mechanism of the mesenchymal-epithelial transition and its relationship with metastatic tumor formation. Mol. Cancer Res.

[24] Liu L (2018). Tiam1 promotes thyroid carcinoma metastasis by modulating EMT via Wnt/β-catenin signaling. Exp. Cell Res..

[25] Wiseman SM (2006). Derangement of the E-cadherin/catenin complex is involved in transformation of differentiated to anaplastic thyroid carcinoma. Am. J. Surg..

[26] Garcia-Rostan G (2001). Beta-catenin dysregulation in thyroid neoplasms: down-regulation, aberrant nuclear expression, and CTNNB1 exon 3 mutations are markers for aggressive tumor phenotypes and poor prognosis. Am. J. Pathol..

[27] Sastre-Perona, A. & Santisteban, P. Role of the Wnt pathway in thyroid cancer. *Front. Endocrinol.* **3**, 10.3389/fendo.2012.00031 (2012). 10.3389/fendo.2012.00031 PMC3355838 22645520

[28] Abbosh PH, Nephew KP (2005). Multiple signaling pathways converge on β-catenin in thyroid cancer. Thyroid.

[29] Garcia-Rostan G (1999). Frequent mutation and nuclear localization of beta-catenin in anaplastic thyroid carcinoma. Cancer Res.

[30] Rao AS (2006). Wnt/β-catenin signaling mediates antineoplastic effects of imatinib mesylate (gleevec) in anaplastic thyroid cancer. J. Clin. Endocrinol. Metab..

[31] Sastre-Perona A, Riesco-Eizaguirre G, Zaballos MA, Santisteban P (2016). beta-catenin signaling is required for RAS-driven thyroid cancer through PI3K activation. Oncotarget.

[32] Padala RR, Karnawat R, Viswanathan SB, Thakkar AV, Das AB (2017). Cancerous perturbations within the ERK, PI3K/Akt, and Wnt/beta-catenin signaling network constitutively activate inter-pathway positive feedback loops. Mol. Biosyst..

[33] Sandsmark E (2017). A novel non-canonical Wnt signature for prostate cancer aggressiveness. Oncotarget.

[34] Viola D (2016). Treatment of advanced thyroid cancer with targeted therapies: ten years of experience. Endocr. Relat. Cancer.

[35] Keutgen XM, Sadowski SM, Kebebew E (2015). Management of anaplastic thyroid cancer. Gland Surg..

[36] Subramanian M (2009). Knockdown of IG20 gene expression renders thyroid cancer cells susceptible to apoptosis. J. Clin. Endocrinol. Metab..

[37] Turner A (2013). MADD knock-down enhances doxorubicin and TRAIL induced apoptosis in breast cancer cells. PLOS ONE.

[38] Li LC (2011). Knockdown of MADD and c-FLIP overcomes resistance to TRAIL-induced apoptosis in ovarian cancer cells. Am. J. Obstet. Gynecol..

[39] Li P (2010). Akt-phosphorylated mitogen-activated kinase-activating death domain protein (MADD) inhibits TRAIL-induced apoptosis by blocking Fas-associated death domain (FADD) association with death receptor 4. J. Biol. Chem..

[40] Lin CS (2018). Role of mitochondrial function in the invasiveness of human colon cancer cells. Oncol. Rep..

[41] Gonzalez DM, Medici D (2014). Signaling mechanisms of the epithelial-mesenchymal transition. Sci. Signal..

[42] Sugitani, I. et al. Prognostic factors and treatment outcomes for anaplastic thyroid carcinoma: ATC Research Consortium of Japan cohort study of 677 patients. *World J. Surg.* **36**, 10.1007/s00268-012-1437-z (2012). 10.1007/s00268-012-1437-z 22311136

[43] Kunstman JW (2015). Characterization of the mutational landscape of anaplastic thyroid cancer via whole-exome sequencing. Human. Mol. Genet..

[44] Tai D (2015). Targeting the WNT signaling pathway in cancer therapeutics. oncologist.

[45] Salvatore G (2007). A cell proliferation and chromosomal instability signature in anaplastic thyroid carcinoma. Cancer Res..

[46] Garg M (2014). Laminin-5γ-2 (LAMC2) is highly expressed in anaplastic thyroid carcinoma and is associated with tumor progression, migration, and invasion by modulating signaling of EGFR. J. Clin. Endocrinol. Metab..

[47] Catalano MG (2012). Histone deacetylase inhibition modulates e-cadherin expression and suppresses migration and invasion of anaplastic thyroid cancer cells. J. Clin. Endocrinol. & Metab..

