# Supplementary figures and images for: Loss of MADD expression inhibits cellular growth and metastasis in anaplastic thyroid cancer
Source: Cell Death Dis. 2019 Feb 13;10(2):145. doi: 10.1038/s41419-019-1351-5 (PMC6374448; doi:10.1038/s41419-019-1351-5)

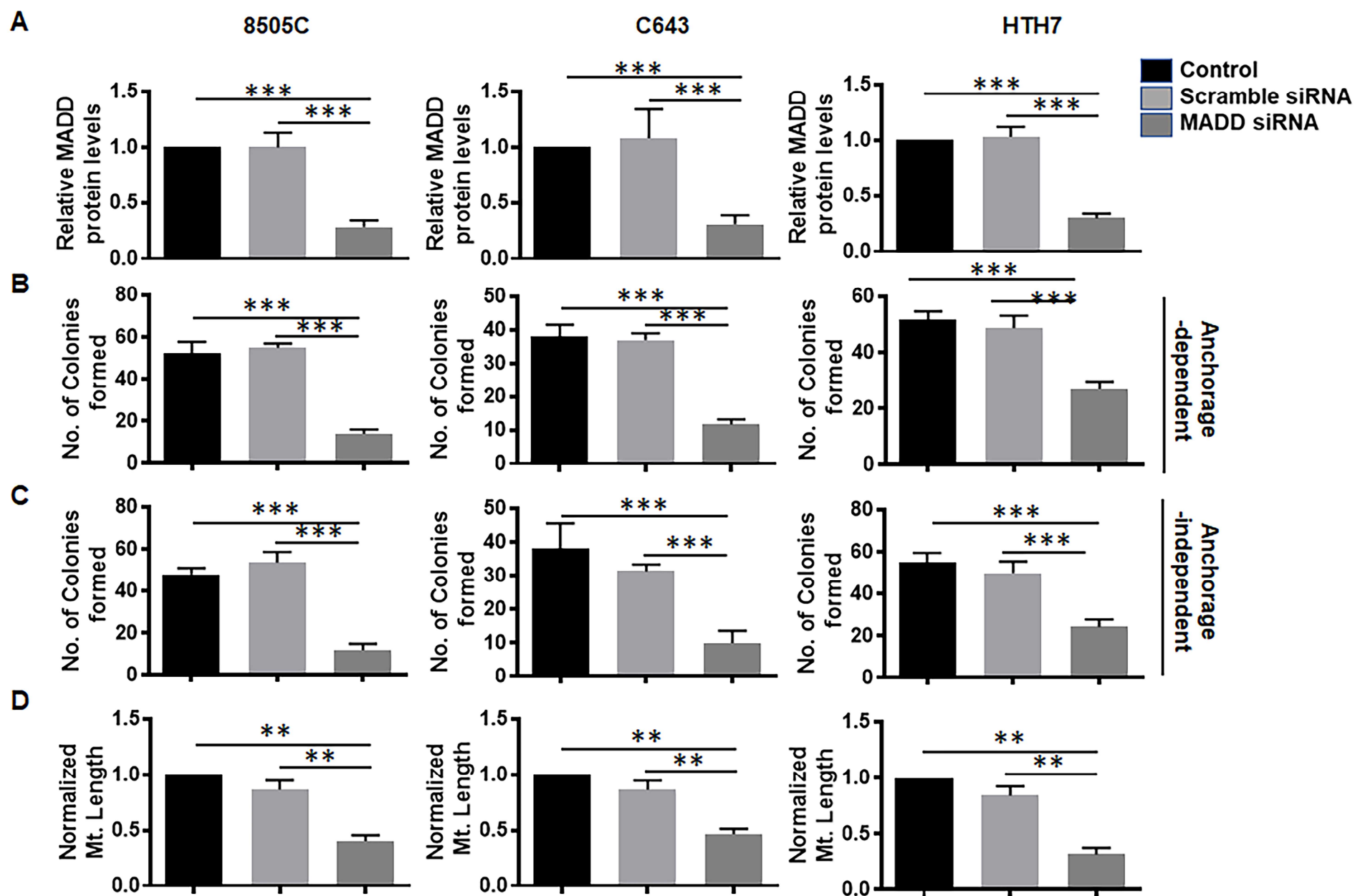

Supplementary Figure S1

Supplement: Supplementary file 1 — Supplementary FIgure S1 [file 41419_2019_1351_MOESM1_ESM.pdf]

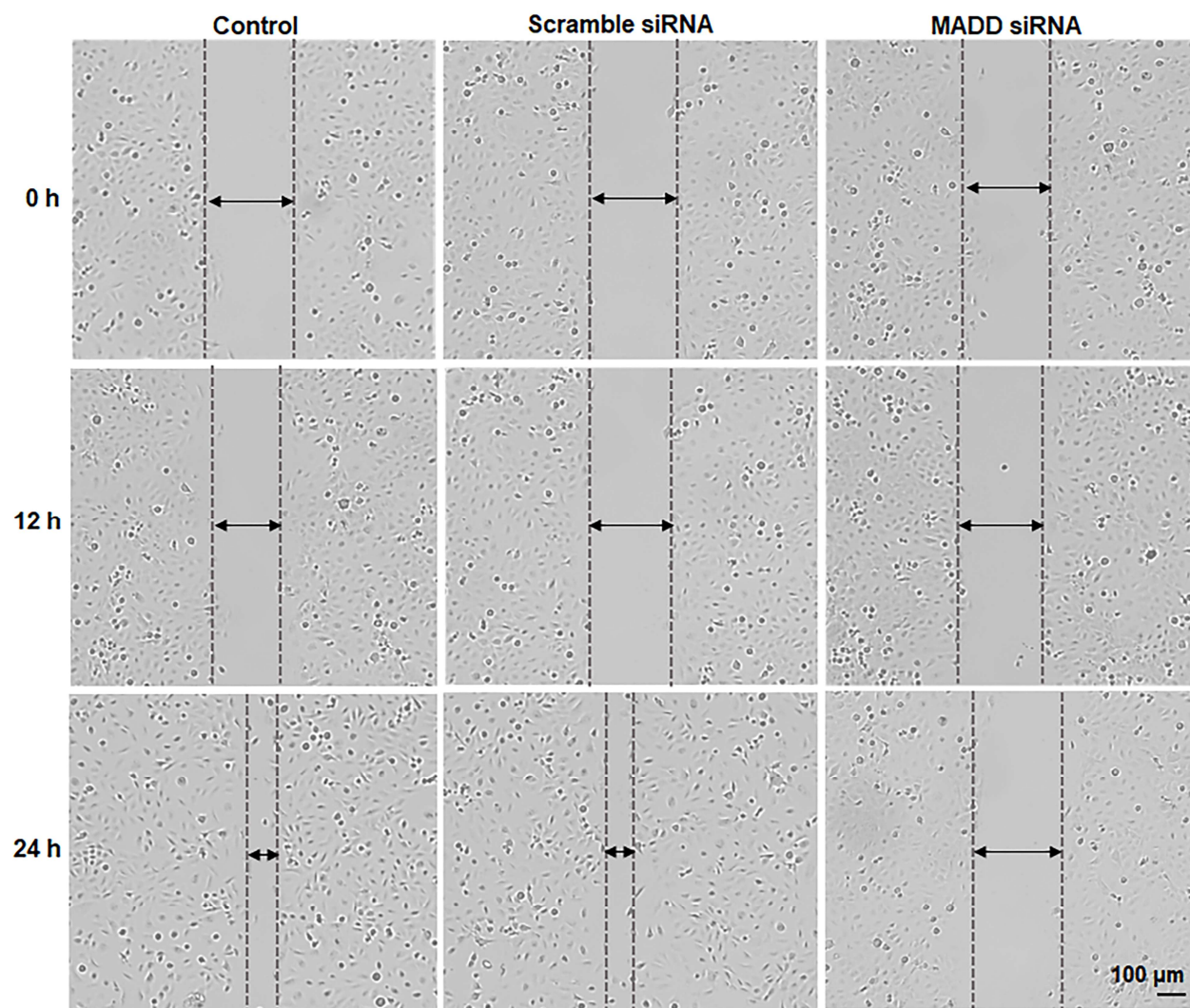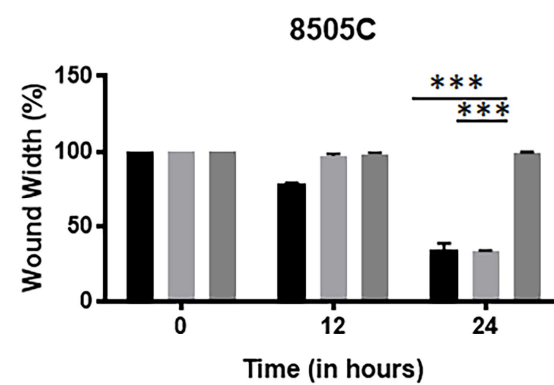

Supplementary Figure S2

Supplement: Supplementary file 2 — Supplementary FIgure S2 [file 41419_2019_1351_MOESM2_ESM.pdf]

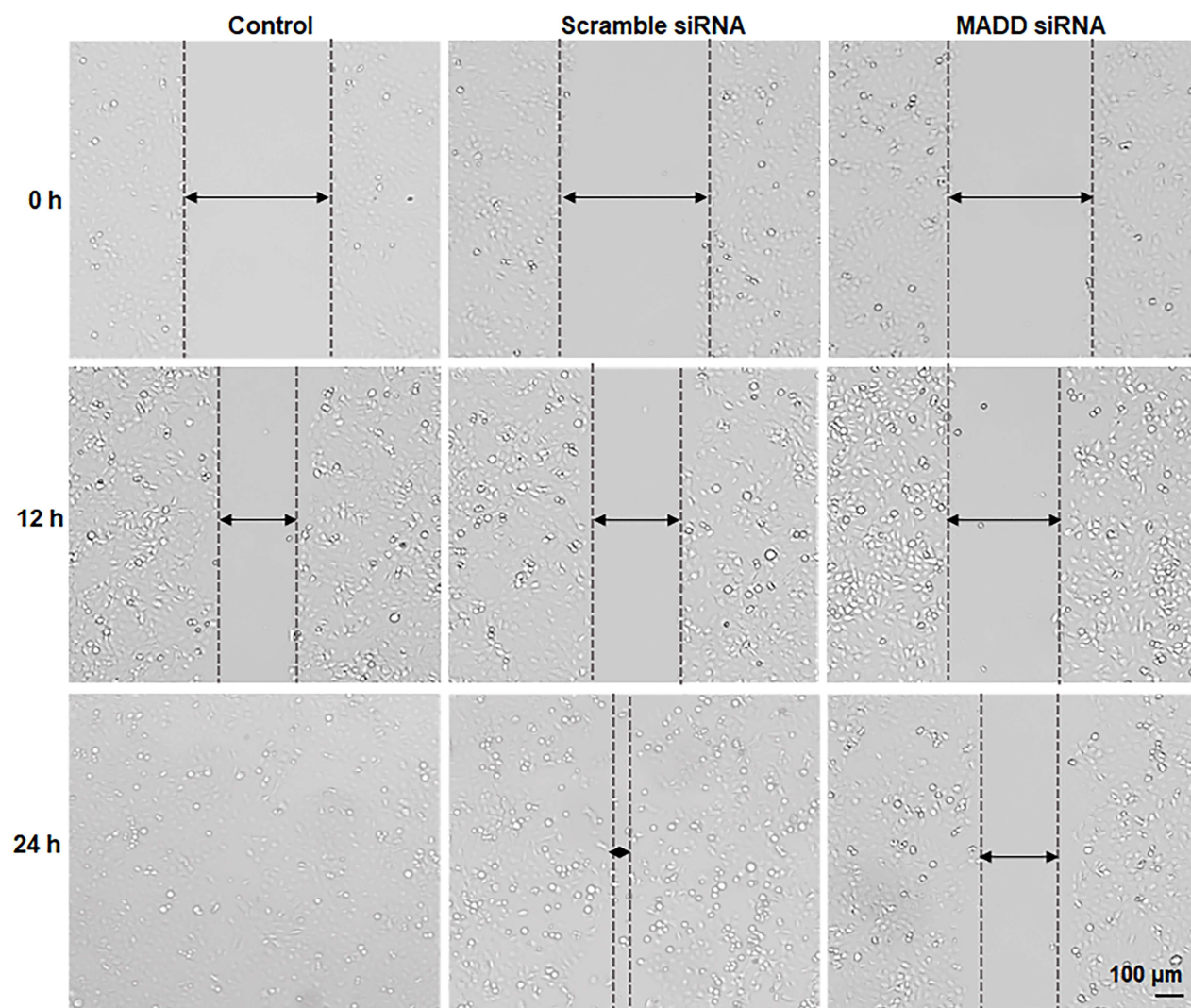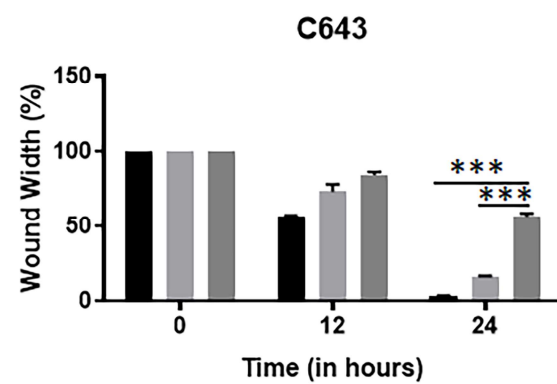

Supplementary Figure S3

Supplement: Supplementary file 3 — Supplementary FIgure S3 [file 41419_2019_1351_MOESM3_ESM.pdf]

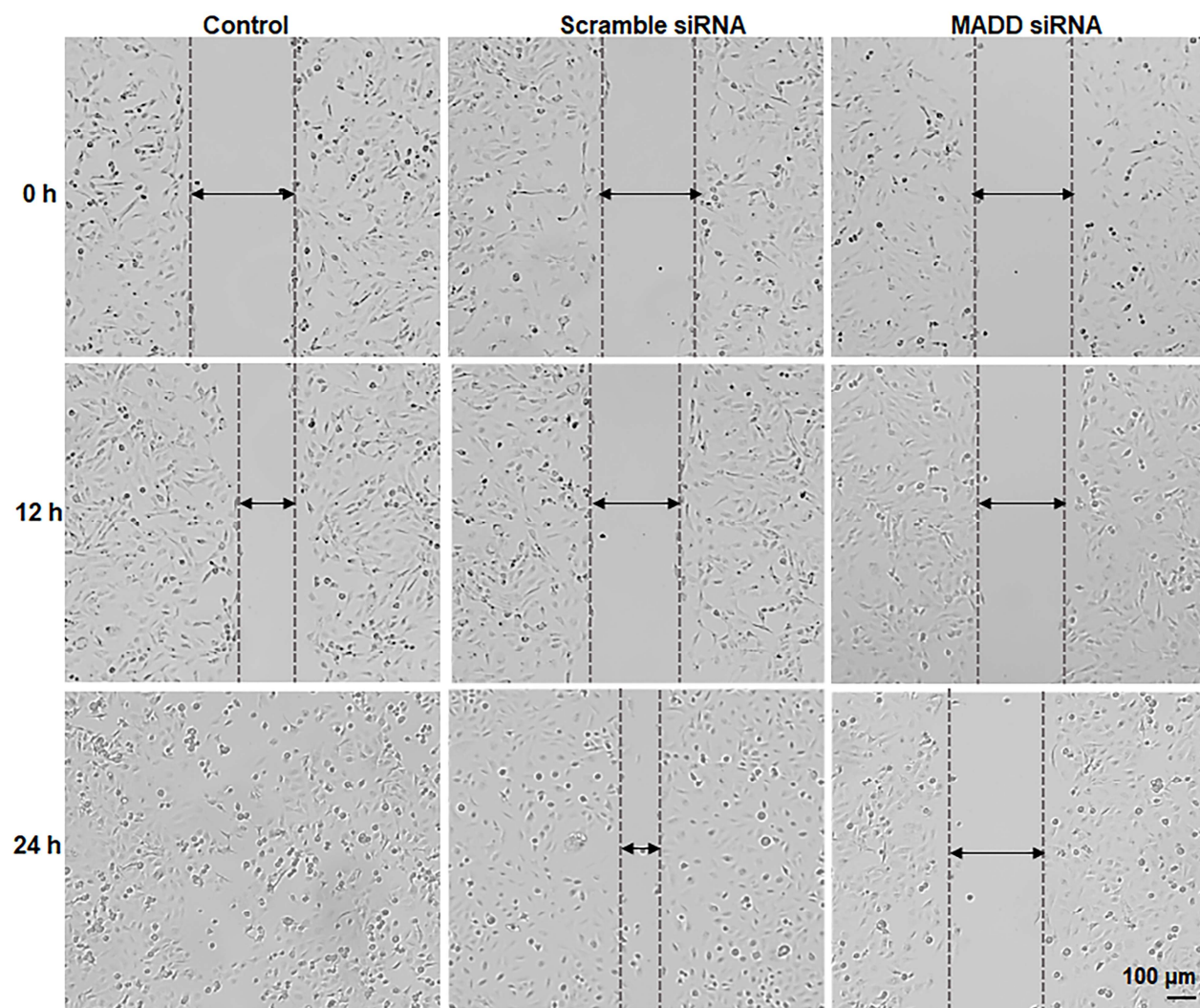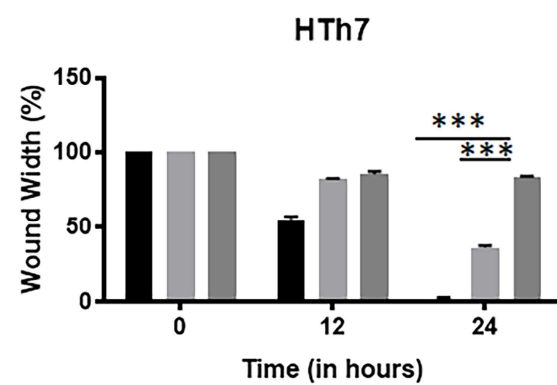

Supplementary Figure S4

Supplement: Supplementary file 4 — Supplementary FIgure S4 [file 41419_2019_1351_MOESM4_ESM.pdf]

**A**

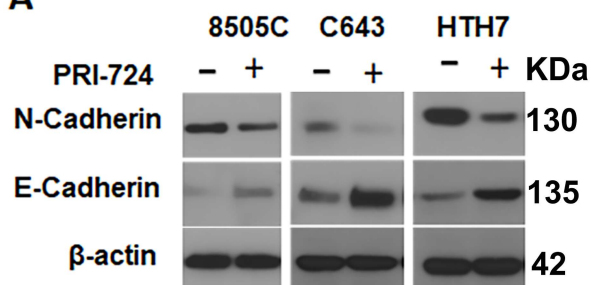

**B**

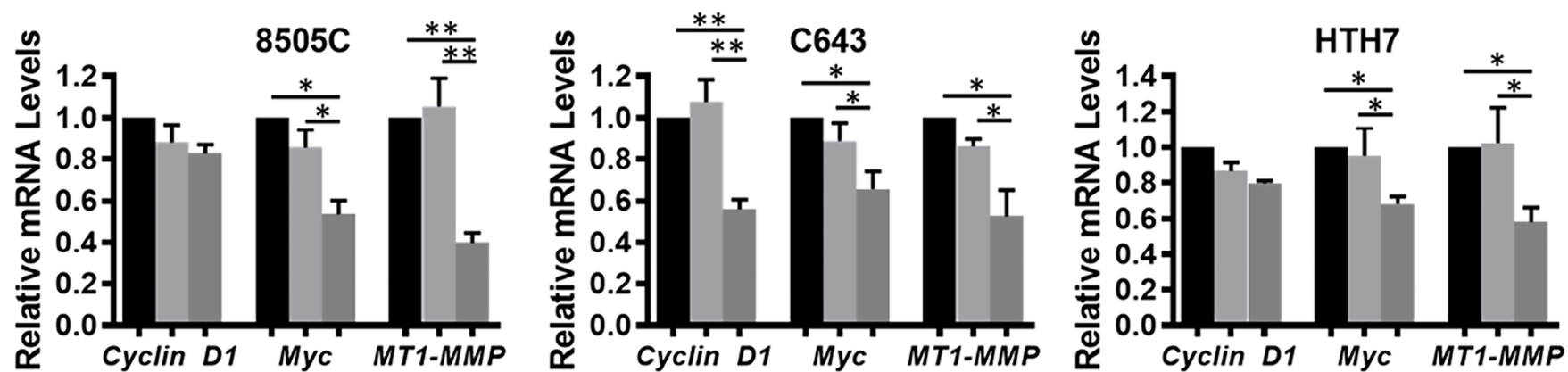

Supplementary Figure S5

Supplement: Supplementary file 5 — Supplementary FIgure S5 [file 41419_2019_1351_MOESM5_ESM.pdf]
