# Supplementary material for: Loss of MADD expression inhibits cellular growth and metastasis in anaplastic thyroid cancer
Source: Cell Death Dis. 2019 Feb 13;10(2):145. doi: 10.1038/s41419-019-1351-5 (PMC6374448; doi:10.1038/s41419-019-1351-5)
Supplement: Supplementary file 6 — supplemental figure legends [file 41419_2019_1351_MOESM6_ESM.docx]

**Supplementary Figure Legends**

*Supplementary Figure S1*: (A) Densitometric analysis of Western blots showing reductions in MADD protein levels upon MADD knock-down in 8505C, C643 and HTH7 cells (B) Quantitative assessment of colony numbers in MADD siRNA-transfected cells versus control and scramble siRNA -transfected cells in 2D colony forming assay; (C) Bar graphs showing number of colonies formed in soft agar plate assay. (D) Representative bar graphs showing relative mitochondrial length comparison between MADD depleted cells and control/scramble siRNA-transfected cells. All assays were performed in triplicates. Values show mean ± SD, **P* < 0.05, ***P* < 0.005, and ***P* < 0.0005.

*Supplementary Figure S2*: Representative microscopic images showing Wound closure at 0, 12 and 24 hours in control, scramble siRNA-transfected and MADD siRNA-transfected 8505C cells. Bar graph depicts the quantitative assessment of the wound length in all groups. Values show mean ± SD, **P* < 0.05, ***P* < 0.005, and ****P* < 0.0005.

*Supplementary Figure S3*: Representative microscopic images showing the effect of MADD silencing on Wound closure at 0, 12 and 24 hours in C643 cells. Bar graph depicts the quantitative assessment of the wound length in all groups. Values show mean ± SD, **P* < 0.05, ***P* < 0.005, and ****P* < 0.0005.

*Supplementary Figure S4*: Representative microphotographs showing the effect of MADD depletion on Wound gap at 0, 12 and 24 hours in HTH7 cells. Bar graph depicts the quantitative assessment of the wound length in all groups. Values show mean ± SD, **P* < 0.05, ***P* < 0.005, and ****P* < 0.0005.

*Supplementary Figure S5:* (A) Western blot analysis showing the correlation between Wnt signaling and EMT activation: Inhibiting Wnt signaling by PRI-724 (25μM) resulted in reduced N-Cadherin and increased E-Cadherin levels in all three ATC cells. (B) Bar graphs representing the effect of MADD knock-down on relative mRNA levels of β-catenin target genes. Values show mean ± SD, **P* < 0.05, ***P* < 0.005, and ****P* < 0.0005.

**Supplementary Table Legends**

*Supplementary Table S1*: List of Antibodies used in this study

*Supplementary Table S2*: Known genetic mutations in ATC cells.
